# Data mining in clinical big data: the frequently used databases, steps, and methodological models

**DOI:** 10.1186/s40779-021-00338-z

**Published:** 2021-08-11

**Authors:** Wen-Tao Wu, Yuan-Jie Li, Ao-Zi Feng, Li Li, Tao Huang, An-Ding Xu, Jun Lyu

**Affiliations:** 1 Department of Clinical Research, The First Affiliated Hospital of Jinan University, Tianhe District, 613 W. Huangpu Avenue, Guangzhou, 510632 Guangdong China; 2 School of Public Health, Xi’an Jiaotong University Health Science Center, Xi’an, 710061 Shaanxi China; 3 Department of Human Anatomy, Histology and Embryology, School of Basic Medical Sciences, Xi’an Jiaotong University Health Science Center, Xi’an, 710061 Shaanxi China; 4 Department of Neurology, The First Affiliated Hospital of Jinan University, Tianhe District, 613 W. Huangpu Avenue, Guangzhou, 510632 Guangdong China

**Keywords:** Clinical big data, Data mining, Machine learning, Medical public database, SEER, NHANES, TCGA, MIMIC

## Abstract

Many high quality studies have emerged from public databases, such as Surveillance, Epidemiology, and End Results (SEER), National Health and Nutrition Examination Survey (NHANES), The Cancer Genome Atlas (TCGA), and Medical Information Mart for Intensive Care (MIMIC); however, these data are often characterized by a high degree of dimensional heterogeneity, timeliness, scarcity, irregularity, and other characteristics, resulting in the value of these data not being fully utilized. Data-mining technology has been a frontier field in medical research, as it demonstrates excellent performance in evaluating patient risks and assisting clinical decision-making in building disease-prediction models. Therefore, data mining has unique advantages in clinical big-data research, especially in large-scale medical public databases. This article introduced the main medical public database and described the steps, tasks, and models of data mining in simple language. Additionally, we described data-mining methods along with their practical applications. The goal of this work was to aid clinical researchers in gaining a clear and intuitive understanding of the application of data-mining technology on clinical big-data in order to promote the production of research results that are beneficial to doctors and patients.

## Background

With the rapid development of computer software/hardware and internet technology, the amount of data has increased at an amazing speed. “Big data” as an abstract concept currently affects all walks of life [1], and although its importance has been recognized, its definition varies slightly from field to field. In the field of computer science, big data refers to a dataset that cannot be perceived, acquired, managed, processed, or served within a tolerable time by using traditional IT and software and hardware tools. Generally, big data refers to a dataset that exceeds the scope of a simple database and data-processing architecture used in the early days of computing and is characterized by high-volume and -dimensional data that is rapidly updated represents a phenomenon or feature that has emerged in the digital age. Across the medical industry, various types of medical data are generated at a high speed, and trends indicate that applying big data in the medical field helps improve the quality of medical care and optimizes medical processes and management strategies [2, 3]. Currently, this trend is shifting from civilian medicine to military medicine. For example, the United States is exploring the potential to use of one of its largest healthcare systems (the Military Healthcare System) to provide healthcare to eligible veterans in order to potentially benefit > 9 million eligible personnel [4]. Another data-management system has been developed to assess the physical and mental health of active-duty personnel, with this expected to yield significant economic benefits to the military medical system [5]. However, in medical research, the wide variety of clinical data and differences between several medical concepts in different classification standards results in a high degree of dimensionality heterogeneity, timeliness, scarcity, and irregularity to existing clinical data [6, 7]. Furthermore, new data analysis techniques have yet to be popularized in medical research [8]. These reasons hinder the full realization of the value of existing data, and the intensive exploration of the value of clinical data remains a challenging problem.

Computer scientists have made outstanding contributions to the application of big data and introduced the concept of data mining to solve difficulties associated with such applications. Data mining (also known as knowledge discovery in databases) refers to the process of extracting potentially useful information and knowledge hidden in a large amount of incomplete, noisy, fuzzy, and random practical application data [9]. Unlike traditional research methods, several data-mining technologies mine information to discover knowledge based on the premise of unclear assumptions (i.e., they are directly applied without prior research design). The obtained information should have previously unknown, valid, and practical characteristics [9]. Data-mining technology does not aim to replace traditional statistical analysis techniques, but it does seek to extend and expand statistical analysis methodologies. From a practical point of view, machine learning (ML) is the main analytical method in data mining, as it represents a method of training models by using data and then using those models for predicting outcomes. Given the rapid progress of data-mining technology and its excellent performance in other industries and fields, it has introduced new opportunities and prospects to clinical big-data research [10]. Large amounts of high quality medical data are available to researchers in the form of public databases, which enable more researchers to participate in the process of medical data mining in the hope that the generated results can further guide clinical practice.

This article provided a valuable overview to medical researchers interested in studying the application of data mining on clinical big data. To allow a clearer understanding of the application of data-mining technology on clinical big data, the second part of this paper introduced the concept of public databases and summarized those commonly used in medical research. In the third part of the paper, we offered an overview of data mining, including introducing an appropriate model, tasks, and processes, and summarized the specific methods of data mining. In the fourth and fifth parts of this paper, we introduced data-mining algorithms commonly used in clinical practice along with specific cases in order to help clinical researchers clearly and intuitively understand the application of data-mining technology on clinical big data. Finally, we discussed the advantages and disadvantages of data mining in clinical analysis and offered insight into possible future applications.

## Overview of common public medical databases

A public database describes a data repository used for research and dedicated to housing data related to scientific research on an open platform. Such databases collect and store heterogeneous and multi-dimensional health, medical, scientific research in a structured form and characteristics of mass/multi-ownership, complexity, and security. These databases cover a wide range of data, including those related to cancer research, disease burden, nutrition and health, and genetics and the environment. Table 1 summarizes the main public medical databases [11–26]. Researchers can apply for access to data based on the scope of the database and the application procedures required to perform relevant medical research.

**Table 1:** Overview of main medical public database

Database	Range	Location	Founded year	Cost	URL	References
Surveillance, Epidemiology, and End Results (SEER)	Tumor	USA	1973	Partially free	https://seer.cancer.gov/	[11]
Medical Information Mart for Intensive Care (MIMIC)	Intensive medical	USA	2001	Free	https://mimic.physionet.org/	[12]
National Health and Nutrition Examination Survey (NHANES)	Children and adults health	USA	1999	Free	https://wwwn.cdc.gov/nchs/nhanes/	[13]
Global Burden of Disease (GBD)	Epidemic trends and burden of disease	Global	1988	Free	http://ghdx.healthdata.org/	[14]
UK Biobank (UKB)	Health-related genetic data and phenotypic data	UK	2006	Partially free	https://www.ukbiobank.ac.uk/	[15]
The Cancer Genome Atlas (TCGA)	Cancer genomics	USA	2006	Free	http://cancergenome.nih.gov/	[16]
Gene Expression Omnibus (GEO)	Sequencing and gene expression	USA	2000	Free	https://www.ncbi.nlm.nih.gov/geo/	[17]
International Cancer Genome Consortium (ICGC)	Cancer genomics	Global	2008	Free	https://dcc.icgc.org/	[18]
China Kadoorie Biobank (CKB)	Chronic diseases	China	2004	Partially free	https://www.ckbiobank.org/site/	[19]
Comparative Toxicogenomics Database (CTD)	Environmental chemicals and human health	USA	2004	Free	http://ctdbase.org/	[20]
Paediatric Intensive Care (PIC)	Paediatric Intensive	China	2010	Free	http://pic.nbscn.org/	[21]
Biologic Specimen and Data Repositories Information Coordinating Center (BioLINCC)	Cardiovascular, pulmonary, and hematological	USA	2009	Free	https://biolincc.nhlbi.nih.gov/	[22]
China Health and Nutrition Survey (CHNS)	Health and nutrition	China	1989	Partially free	http://www.cpc.unc.edu/projects/china	[23]
China Health and Retirement Longitudinal Study (CHARLS)	Ageing and health	China	2011	Free	http://charls.pku.edu.cn/	[24]
eICU Collaborative Research Database (eICU-CRD)	Intensive medical	USA	2018	Free	https://eicu-crd.mit.edu/	[25]
Health and Retirement Study (HRS)	Aging health and social support	Global	1992	Free	https://hrs.isr.umich.edu/	[26]

## Data mining: an overview

Data mining is a multidisciplinary field at the intersection of database technology, statistics, ML, and pattern recognition that profits from all these disciplines [27]. Although this approach is not yet widespread in the field of medical research, several studies have demonstrated the promise of data mining in building disease-prediction models, assessing patient risk, and helping physicians make clinical decisions [28–31].

### Data-mining models

Data-mining has two kinds of models: descriptive and predictive. Predictive models are used to predict unknown or future values of other variables of interest, whereas descriptive models are often used to find patterns that describe data that can be interpreted by humans [32].

### Data-mining tasks

A model is usually implemented by a task, with the goal of description being to generalize patterns of potential associations in the data. Therefore, using a descriptive model usually results in a few collections with the same or similar attributes. Prediction mainly refers to estimation of the variable value of a specific attribute based on the variable values of other attributes, including classification and regression [33].

### Data-mining methods

After defining the data-mining model and task, the data mining methods required to build the approach based on the discipline involved are then defined. The data-mining method depends on whether or not dependent variables (labels) are present in the analysis. Predictions with dependent variables (labels) are generated through supervised learning, which can be performed by the use of linear regression, generalized linear regression, a proportional hazards model (the Cox regression model), a competitive risk model, decision trees, the random forest (RF) algorithm, and support vector machines (SVMs). In contrast, unsupervised learning involves no labels. The learning model infers some internal data structure. Common unsupervised learning methods include principal component analysis (PCA), association analysis, and clustering analysis.

## Data-mining algorithms for clinical big data

Data mining based on clinical big data can produce effective and valuable knowledge, which is essential for accurate clinical decision-making and risk assessment [34]. Data-mining algorithms enable realization of these goals.

### Supervised learning

A concept often mentioned in supervised learning is the partitioning of datasets. To prevent overfitting of a model, a dataset can generally be divided into two or three parts: a training set, validation set, and test set. Ripley [35] defined these parts as a set of examples used for learning and used to fit the parameters (i.e., weights) of the classifier, a set of examples used to tune the parameters (i.e., architecture) of a classifier, and a set of examples used only to assess the performance (generalized) of a fully-specified classifier, respectively. Briefly, the training set is used to train the model or determine the model parameters, the validation set is used to perform model selection, and the test set is used to verify model performance. In practice, data are generally divided into training and test sets, whereas the verification set is less involved. It should be emphasized that the results of the test set do not guarantee model correctness but only show that similar data can obtain similar results using the model. Therefore, the applicability of a model should be analysed in combination with specific problems in the research. Classical statistical methods, such as linear regression, generalized linear regression, and a proportional risk model, have been widely used in medical research. Notably, most of these classical statistical methods have certain data requirements or assumptions; however, in face of complicated clinical data, assumptions about data distribution are difficult to make. In contrast, some ML methods (algorithmic models) make no assumptions about the data and cross-verify the results; thus, they are likely to be favoured by clinical researchers [36]. For these reasons, this chapter focuses on ML methods that do not require assumptions about data distribution and classical statistical methods that are used in specific situations.

#### Decision tree

A decision tree is a basic classification and regression method that generates a result similar to the tree structure of a flowchart, where each tree node represents a test on an attribute, each branch represents the output of an attribute, each leaf node (decision node) represents a class or class distribution, and the topmost part of the tree is the root node [37]. The decision tree model is called a classification tree when used for classification and a regression tree when used for regression. Studies have demonstrated the utility of the decision tree model in clinical applications. In a study on the prognosis of breast cancer patients, a decision tree model and a classical logistic regression model were constructed, respectively, with the predictive performance of the different models indicating that the decision tree model showed stronger predictive power when using real clinical data [38]. Similarly, the decision tree model has been applied to other areas of clinical medicine, including diagnosis of kidney stones [39], predicting the risk of sudden cardiac arrest [40], and exploration of the risk factors of type II diabetes [41]. A common feature of these studies is the use of a decision tree model to explore the interaction between variables and classify subjects into homogeneous categories based on their observed characteristics. In fact, because the decision tree accounts for the strong interaction between variables, it is more suitable for use with decision algorithms that follow the same structure [42]. In the construction of clinical prediction models and exploration of disease risk factors and patient prognosis, the decision tree model might offer more advantages and practical application value than some classical algorithms. Although the decision tree has many advantages, it recursively separates observations into branches to construct a tree; therefore, in terms of data imbalance, the precision of decision tree models needs improvement.

#### The RF method

The RF algorithm was developed as an application of an ensemble-learning method based on a collection of decision trees. The bootstrap method [43] is used to randomly retrieve sample sets from the training set, with decision trees generated by the bootstrap method constituting a “random forest” and predictions based on this derived from an ensemble average or majority vote. The biggest advantage of the RF method is that the random sampling of predictor variables at each decision tree node decreases the correlation among the trees in the forest, thereby improving the precision of ensemble predictions [44]. Given that a single decision tree model might encounter the problem of overfitting [45], the initial application of RF minimizes overfitting in classification and regression and improves predictive accuracy [44]. Taylor et al. [46] highlighted the potential of RF in correctly differentiating in-hospital mortality in patients experiencing sepsis after admission to the emergency department. Nowhere in the healthcare system is the need more pressing to find methods to reduce uncertainty than in the fast, chaotic environment of the emergency department. The authors demonstrated that the predictive performance of the RF method was superior to that of traditional emergency medicine methods and the methods enabled evaluation of more clinical variables than traditional modelling methods, which subsequently allowed the discovery of clinical variables not expected to be of predictive value or which otherwise would have been omitted as a rare predictor [46]. Another study based on the Medical Information Mart for Intensive Care (MIMIC) II database [47] found that RF had excellent predictive power regarding intensive care unit (ICU) mortality [48]. These studies showed that the application of RF to big data stored in the hospital healthcare system provided a new data-driven method for predictive analysis in critical care. Additionally, random survival forests have recently been developed to analyse survival data, especially right-censored survival data [49, 50], which can help researchers conduct survival analyses in clinical oncology and help develop personalized treatment regimens that benefit patients [51].

#### SVMs

The SVM is a relatively new classification or prediction method developed by Cortes and Vapnik and represents a data-driven approach that does not require assumptions about data distribution [52]. The core purpose of an SVM is to identify a separation boundary (called a hyperplane) to help classify cases; thus, the advantages of SVMs are obvious when classifying and predicting cases based on high dimensional data or data with a small sample size [53, 54].

In a study of drug compliance in patients with heart failure, researchers used an SVM to build a predictive model for patient compliance in order to overcome the problem of a large number of input variables relative to the number of available observations [55]. Additionally, the mechanisms of certain chronic and complex diseases observed in clinical practice remain unclear, and many risk factors, including gene–gene interactions and gene-environment interactions, must be considered in the research of such diseases [55, 56]. SVMs are capable of addressing these issues. Yu et al. [54] applied an SVM for predicting diabetes onset based on data from the National Health and Nutrition Examination Survey (NHANES). Furthermore, these models have strong discrimination ability, making SVMs a promising classification approach for detecting individuals with chronic and complex diseases. However, a disadvantage of SVMs is that when the number of observation samples is large, the method becomes time- and resource-intensive, which is often highly inefficient.

#### Competitive risk model

Kaplan–Meier marginal regression and the Cox proportional hazards model are widely used in survival analysis in clinical studies. Classical survival analysis usually considers only one endpoint, such as the impact of patient survival time. However, in clinical medical research, multiple endpoints usually coexist, and these endpoints compete with one another to generate competitive risk data [57]. In the case of multiple endpoint events, the use of a single endpoint-analysis method can lead to a biased estimation of the probability of endpoint events due to the existence of competitive risks [58]. The competitive risk model is a classical statistical model based on the hypothesis of data distribution. Its main advantage is its accurate estimation of the cumulative incidence of outcomes for right-censored survival data with multiple endpoints [59]. In data analysis, the cumulative risk rate is estimated using the cumulative incidence function in single-factor analysis, and Gray’s test is used for between-group comparisons [60].

Multifactor analysis uses the Fine-Gray and cause-specific (CS) risk models to explore the cumulative risk rate [61]. The difference between the Fine-Gray and CS models is that the former is applicable to establishing a clinical prediction model and predicting the risk of a single endpoint of interest [62], whereas the latter is suitable for answering etiological questions, where the regression coefficient reflects the relative effect of covariates on the increased incidence of the main endpoint in the target event-free risk set [63]. Currently, in databases with CS records, such as Surveillance, Epidemiology, and End Results (SEER), competitive risk models exhibit good performance in exploring disease-risk factors and prognosis [64]. A study of prognosis in patients with oesophageal cancer from SEER showed that Cox proportional risk models might misestimate the effects of age and disease location on patient prognosis, whereas competitive risk models provide more accurate estimates of factors affecting patient prognosis [65]. In another study of the prognosis of penile cancer patients, researchers found that using a competitive risk model was more helpful in developing personalized treatment plans [66].

### Unsupervised learning

In many data-analysis processes, the amount of usable identified data is small, and identifying data is a tedious process [67]. Unsupervised learning is necessary to judge and categorize data according to similarities, characteristics, and correlations and has three main applications: data clustering, association analysis, and dimensionality reduction. Therefore, the unsupervised learning methods introduced in this section include clustering analysis, association rules, and PCA.

#### Clustering analysis

The classification algorithm needs to “know” information concerning each category in advance, with all of the data to be classified having corresponding categories. When the above conditions cannot be met, cluster analysis can be applied to solve the problem [68]. Clustering places similar objects into different categories or subsets through the process of static classification. Consequently, objects in the same subset have similar properties. Many kinds of clustering techniques exist. Here, we introduced the four most commonly used clustering techniques.

##### Partition clustering

The core idea of this clustering method regards the centre of the data point as the centre of the cluster. The k-means method [69] is a representative example of this technique. The k-means method takes *n* observations and an integer, *k*, and outputs a partition of the *n* observations into *k* sets such that each observation belongs to the cluster with the nearest mean [70]. The k-means method exhibits low time complexity and high computing efficiency but has a poor processing effect on high dimensional data and cannot identify nonspherical clusters.

##### Hierarchical clustering

The hierarchical clustering algorithm decomposes a dataset hierarchically to facilitate the subsequent clustering [71]. Common algorithms for hierarchical clustering include BIRCH [72], CURE [73], and ROCK [74]. The algorithm starts by treating every point as a cluster, with clusters grouped according to closeness. When further combinations result in unexpected results under multiple causes or only one cluster remains, the grouping process ends. This method has wide applicability, and the relationship between clusters is easy to detect; however, the time complexity is high [75].

##### Clustering according to density

The density algorithm takes areas presenting a high degree of data density and defines these as belonging to the same cluster [76]. This method aims to find arbitrarily-shaped clusters, with the most representative algorithm being DBSCAN [77]. In practice, DBSCAN does not need to input the number of clusters to be partitioned and can handle clusters of various shapes; however, the time complexity of the algorithm is high. Furthermore, when data density is irregular, the quality of the clusters decreases; thus, DBSCAN cannot process high dimensional data [75].

##### Clustering according to a grid

Neither partition nor hierarchical clustering can identify clusters with nonconvex shapes. Although a dimension-based algorithm can accomplish this task, the time complexity is high. To address this problem, data-mining researchers proposed grid-based algorithms that changed the original data space into a grid structure of a certain size. A representative algorithm is STING, which divides the data space into several square cells according to different resolutions and clusters the data of different structure levels [78]. The main advantage of this method is its high processing speed and its exclusive dependence on the number of units in each dimension of the quantized space.

In clinical studies, subjects tend to be actual patients. Although researchers adopt complex inclusion and exclusion criteria before determining the subjects to be included in the analyses, heterogeneity among different patients cannot be avoided [79, 80]. The most common application of cluster analysis in clinical big data is in classifying heterogeneous mixed groups into homogeneous groups according to the characteristics of existing data (i.e., “subgroups” of patients or observed objects are identified) [81, 82]. This new information can then be used in the future to develop patient-oriented medical-management strategies. Docampo et al. [81] used hierarchical clustering to reduce heterogeneity and identify subgroups of clinical fibromyalgia, which aided the evaluation and management of fibromyalgia. Additionally, Guo et al. [83] used k-means clustering to divide patients with essential hypertension into four subgroups, which revealed that the potential risk of coronary heart disease differed between different subgroups. On the other hand, density- and grid-based clustering algorithms have mostly been used to process large numbers of images generated in basic research and clinical practice, with current studies focused on developing new tools to help clinical research and practices based on these technologies [84, 85]. Cluster analysis will continue to have extensive application prospects along with the increasing emphasis on personalized treatment.

#### Association rules

Association rules discover interesting associations and correlations between item sets in large amounts of data. These rules were first proposed by Agrawal et al. [86] and applied to analyse customer buying habits to help retailers create sales plans. Data-mining based on association rules identifies association rules in a two-step process: 1) all high frequency items in the collection are listed and 2) frequent association rules are generated based on the high frequency items [87]. Therefore, before association rules can be obtained, sets of frequent items must be calculated using certain algorithms. The Apriori algorithm is based on the a priori principle of finding all relevant adjustment items in a database transaction that meet a minimum set of rules and restrictions or other restrictions [88]. Other algorithms are mostly variants of the Apriori algorithm [64]. The Apriori algorithm must scan the entire database every time it scans the transaction; therefore, algorithm performance deteriorates as database size increases [89], making it potentially unsuitable for analysing large databases. The frequent pattern (FP) growth algorithm was proposed to improve efficiency. After the first scan, the FP algorithm compresses the frequency set in the database into a FP tree while retaining the associated information and then mines the conditional libraries separately [90]. Association-rule technology is often used in medical research to identify association rules between disease risk factors (i.e., exploration of the joint effects of disease risk factors and combinations of other risk factors). For example, Li et al. [91] used the association-rule algorithm to identify the most important stroke risk factor as atrial fibrillation, followed by diabetes and a family history of stroke. Based on the same principle, association rules can also be used to evaluate treatment effects and other aspects. For example, Guo et al. [92] used the FP algorithm to generate association rules and evaluate individual characteristics and treatment effects of patients with diabetes, thereby reducing the readability rate of patients with diabetes. Association rules reveal a connection between premises and conclusions; however, the reasonable and reliable application of information can only be achieved through validation by experienced medical professionals and through extensive causal research [92].

#### PCA

PCA is a widely used data-mining method that aims to reduce data dimensionality in an interpretable way while retaining most of the information present in the data [93, 94]. The main purpose of PCA is descriptive, as it requires no assumptions about data distribution and is, therefore, an adaptive and exploratory method. During the process of data analysis, the main steps of PCA include standardization of the original data, calculation of a correlation coefficient matrix, calculation of eigenvalues and eigenvectors, selection of principal components, and calculation of the comprehensive evaluation value. PCA does not often appear as a separate method, as it is often combined with other statistical methods [95]. In practical clinical studies, the existence of multicollinearity often leads to deviation from multivariate analysis. A feasible solution is to construct a regression model by PCA, which replaces the original independent variables with each principal component as a new independent variable for regression analysis, with this most commonly seen in the analysis of dietary patterns in nutritional epidemiology [96]. In a study of socioeconomic status and child-developmental delays, PCA was used to derive a new variable (the household wealth index) from a series of household property reports and incorporate this new variable as the main analytical variable into the logistic regression model [97]. Additionally, PCA can be combined with cluster analysis. Burgel et al. [98] used PCA to transform clinical data to address the lack of independence between existing variables used to explore the heterogeneity of different subtypes of chronic obstructive pulmonary disease. Therefore, in the study of subtypes and heterogeneity of clinical diseases, PCA can eliminate noisy variables that can potentially corrupt the cluster structure, thereby increasing the accuracy of the results of clustering analysis [98, 99].

## The data-mining process and examples of its application using common public databases

Open-access databases have the advantages of large volumes of data, wide data coverage, rich data information, and a cost-efficient method of research, making them beneficial to medical researchers. In this chapter, we introduced the data-mining process and methods and their application in research based on examples of utilizing public databases and data-mining algorithms.

### The data-mining process

**Fig. 1:**
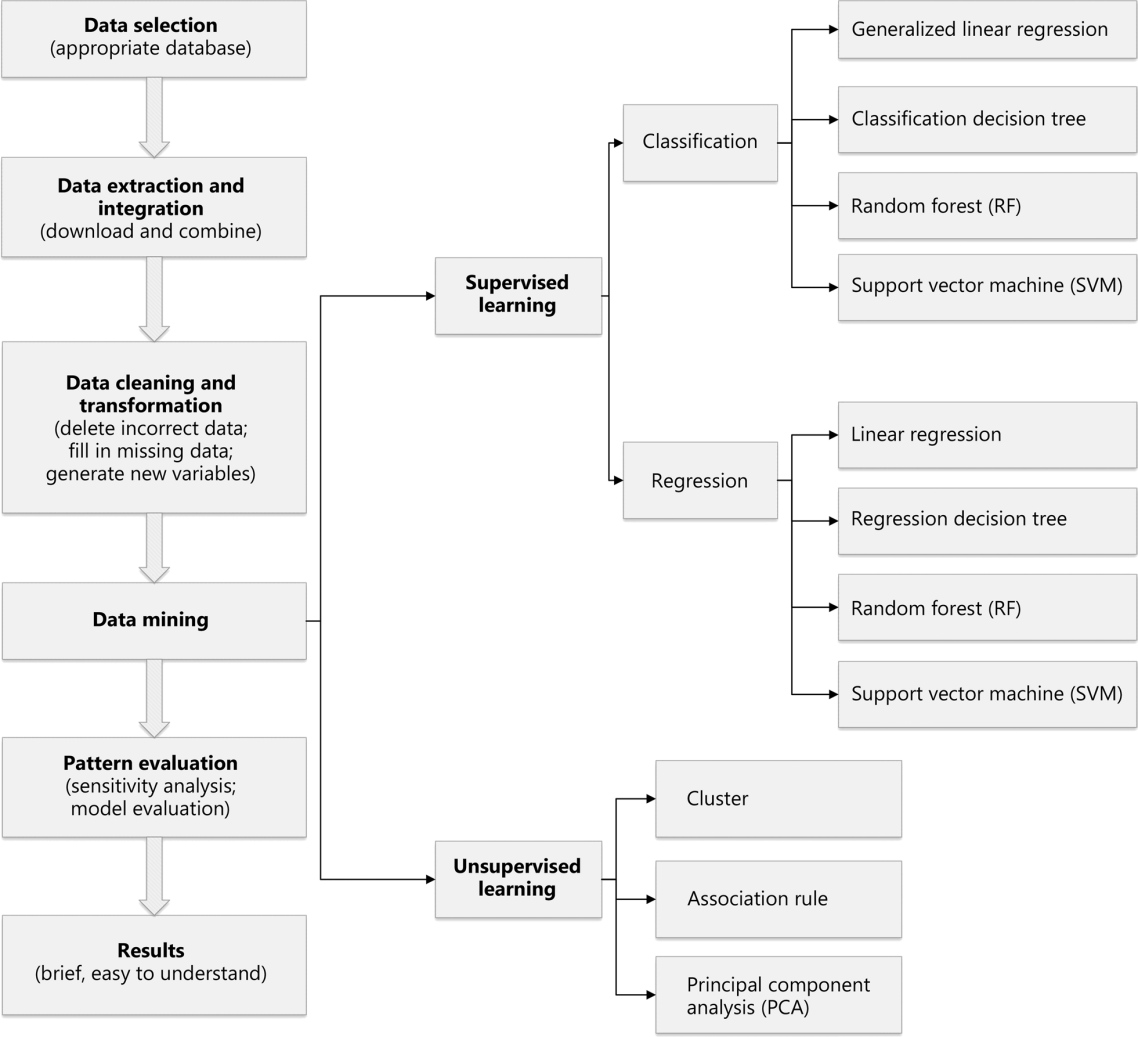
The steps of data mining in medical public database

Figure 1 shows a series of research concepts. The data-mining process is divided into several steps: (1) database selection according to the research purpose; (2) data extraction and integration, including downloading the required data and combining data from multiple sources; (3) data cleaning and transformation, including removal of incorrect data, filling in missing data, generating new variables, converting data format, and ensuring data consistency; (4) data mining, involving extraction of implicit relational patterns through traditional statistics or ML; (5) pattern evaluation, which focuses on the validity parameters and values of the relationship patterns of the extracted data; and (6) assessment of the results, involving translation of the extracted data-relationship model into comprehensible knowledge made available to the public.

### Examples of data-mining applied using public databases

#### Establishment of warning models for the early prediction of disease

A previous study identified sepsis as a major cause of death in ICU patients [100]. The authors noted that the predictive model developed previously used a limited number of variables, and that model performance required improvement. The data-mining process applied to address these issues was, as follows: (1) data selection using the MIMIC III database; (2) extraction and integration of three types of data, including multivariate features (demographic information and clinical biochemical indicators), time series data (temperature, blood pressure, and heart rate), and clinical latent features (various scores related to disease); (3) data cleaning and transformation, including fixing irregular time series measurements, estimating missing values, deleting outliers, and addressing data imbalance; (4) data mining through the use of logical regression, generation of a decision tree, application of the RF algorithm, an SVM, and an ensemble algorithm (a combination of multiple classifiers) to established the prediction model; (5) pattern evaluation using sensitivity, precision, and the area under the receiver operating characteristic curve to evaluate model performance; and (6) evaluation of the results, in this case the potential to predicting the prognosis of patients with sepsis and whether the model outperformed current scoring systems.

#### Exploring prognostic risk factors in cancer patients

Wu et al. [101] noted that traditional survival-analysis methods often ignored the influence of competitive risk events, such as suicide and car accident, on outcomes, leading to deviations and misjudgements in estimating the effect of risk factors. They used the SEER database, which offers cause-of-death data for cancer patients, and a competitive risk model to address this problem according to the following process: (1) data were obtained from the SEER database; (2) demography, clinical characteristics, treatment modality, and cause of death of cecum cancer patients were extracted from the database; (3) patient data were deleted when there were no demographic, clinical, therapeutic, or cause-of-death variables; (4) Cox regression and two kinds of competitive risk models were applied for survival analysis; (5) the results were compared between three different models; and (6) the results revealed that for survival data with multiple endpoints, the competitive risk model was more favourable.

#### Derivation of dietary patterns

A study by Martínez Steele et al. [102] applied PCA for nutritional epidemiological analysis to determine dietary patterns and evaluate the overall nutritional quality of the population based on those patterns. Their process involved the following: (1) data were extracted from the NHANES database covering the years 2009–2010; (2) demographic characteristics and two 24 h dietary recall interviews were obtained; (3) data were weighted and excluded based on subjects not meeting specific criteria; (4) PCA was used to determine dietary patterns in the United States population, and Gaussian regression and restricted cubic splines were used to assess associations between ultra-processed foods and nutritional balance; (5) eigenvalues, scree plots, and the interpretability of the principal components were reviewed to screen and evaluate the results; and (6) the results revealed a negative association between ultra-processed food intake and overall dietary quality. Their findings indicated that a nutritionally balanced eating pattern was characterized by a diet high in fibre, potassium, magnesium, and vitamin C intake along with low sugar and saturated fat consumption.

## Conclusion

The use of “big data” has changed multiple aspects of modern life, with its use combined with data-mining methods capable of improving the status quo [86]. The aim of this study was to aid clinical researchers in understanding the application of data-mining technology on clinical big data and public medical databases to further their research goals in order to benefit clinicians and patients. The examples provided offer insight into the data-mining process applied for the purposes of clinical research. Notably, researchers have raised concerns that big data and data-mining methods were not a perfect fit for adequately replicating actual clinical conditions, with the results potentially capable of misleading doctors and patients [86]. Therefore, given the rate at which new technologies and trends progress, it is necessary to maintain a positive attitude concerning their potential impact while remaining cautious in examining the results provided by their application.

In the future, the healthcare system will need to utilize increasingly larger volumes of big data with higher dimensionality. The tasks and objectives of data analysis will also have higher demands, including higher degrees of visualization, results with increased accuracy, and stronger real-time performance. As a result, the methods used to mine and process big data will continue to improve. Furthermore, to increase the formality and standardization of data-mining methods, it is possible that a new programming language specifically for this purpose will need to be developed, as well as novel methods capable of addressing unstructured data, such as graphics, audio, and text represented by handwriting. In terms of application, the development of data-management and disease-screening systems for large-scale populations, such as the military, will help determine the best interventions and formulation of auxiliary standards capable of benefitting both cost-efficiency and personnel. Data-mining technology can also be applied to hospital management in order to improve patient satisfaction, detect medical-insurance fraud and abuse, and reduce costs and losses while improving management efficiency. Currently, this technology is being applied for predicting patient disease, with further improvements resulting in the increased accuracy and speed of these predictions. Moreover, it is worth noting that technological development will concomitantly require higher quality data, which will be a prerequisite for accurate application of the technology.

Finally, the ultimate goal of this study was to explain the methods associated with data mining and commonly used to process clinical big data. This review will potentially promote further study and aid doctors and patients.

## Abbreviations

BioLINCC: Biologic Specimen and Data Repositories Information Coordinating Center
CHARLS: China Health and Retirement Longitudinal Study
CHNS: China Health and Nutrition Survey
CKB: China Kadoorie Biobank
CS: Cause-specific risk
CTD: Comparative Toxicogenomics Database
eICU-CRD: EICU Collaborative Research Database
FP: Frequent pattern
GBD: Global burden of disease
GEO: Gene expression omnibus
HRS: Health and Retirement Study
ICGC: International Cancer Genome Consortium
MIMIC: Medical Information Mart for Intensive Care
ML: Machine learning
NHANES: National Health and Nutrition Examination Survey
PCA: Principal component analysis
PIC: Paediatric intensive care
RF: Random forest
SEER: Surveillance, epidemiology, and end results
SVM: Support vector machine
TCGA: The Cancer Genome Atlas
UKB: UK Biobank
